# Water-Induced Changes in Experimental Resin Composites Functionalized with Conventional (45S5) and Customized Bioactive Glass

**DOI:** 10.3390/jfb14060298

**Published:** 2023-05-27

**Authors:** Alen Muradbegovic, Matej Par, Vlatko Panduric, Paula Zugec, Tobias T. Tauböck, Thomas Attin, Zrinka Tarle, Danijela Marovic

**Affiliations:** 1 Muradbegović Dental Clinic, Malkočeva 3, 75000 Tuzla, Bosnia and Herzegovina; muradbegovic.alen@gmail.com; 2 Department of Endodontics and Restorative Dentistry, School of Dental Medicine, University of Zagreb, Gunduliceva 5, 10000 Zagreb, Croatia; vpanduric@sfzg.hr (V.P.); pzugec@sfzg.hr (P.Z.); tarle@sfzg.hr (Z.T.); marovic@sfzg.hr (D.M.); 3 Department of Conservative and Preventive Dentistry, Center of Dental Medicine, University of Zurich, Plattenstrasse 11, 8032 Zurich, Switzerland; tobias.tauboeck@zzm.uzh.ch (T.T.T.); thomas.attin@zzm.uzh.ch (T.A.)

**Keywords:** bioactive glass, experimental resin composites, microhardness, calcium phosphate, water sorption, solubility

## Abstract

The aim of the study was to evaluate microhardness, mass changes during 1-year water immersion, water sorption/solubility, and calcium phosphate precipitation of experimental composites functionalized with 5–40 wt% of two types of bioactive glass (BG): 45S5 or a customized low-sodium fluoride-containing formulation. Vickers microhardness was evaluated after simulated aging (water storage and thermocycling), water sorption and solubility were tested according to ISO 4049, and calcium phosphate precipitation was studied by scanning electron microscopy, energy dispersive X-ray spectroscopy, and Fourier-transform infrared spectroscopy. For the composites containing BG 45S5, a significant reduction in microhardness was observed with increasing BG amount. In contrast, 5 wt% of the customized BG resulted in statistically similar microhardness to the control material, while higher BG amounts (20 and 40 wt%) resulted in a significant improvement in microhardness. Water sorption was more pronounced for composites containing BG 45S5, increasing 7-fold compared to the control material, while the corresponding increase for the customized BG was only 2-fold. Solubility increased with higher amounts of BG, with an abrupt increase at 20 and 40 wt% of BG 45S5. Calcium phosphate was precipitated by all composites with BG amounts of 10 wt% or more. The improved properties of the composites functionalized with the customized BG indicate better mechanical, chemical, and dimensional stability without compromising the potential for calcium phosphate precipitation.

## 1. Introduction

A variety of ion-releasing and potentially remineralizing dental composite materials have been experimentally developed and investigated as a means of preventing secondary caries [1]. Ion-releasing composites based on bioactive glasses (BGs) [2] have gained popularity in the last decade due to their ability to release various ions [3,4], increase pH [5], and possibly even seal marginal gaps by apatite precipitation [6].

A 2022 World Dental Federation (FDI) policy statement emphasizes the need to ensure that the addition of reactive fillers to restorative materials to achieve an anti-cariogenic effect does not compromise the primary function of restorative materials, namely the replacement of tooth structure [7]. To meet this requirement, materials should maintain stable mechanical properties throughout their lifetime exposure to water. For composites functionalized with reactive fillers, a moderate reduction in mechanical properties due to exposure to water is inevitable, as components of the material dissolve and diffuse into the surrounding aqueous medium [8,9]. The dissolution of the reactive fillers and the plasticization of the resin matrix lead to a weakening of the material structure, which is followed by water diffusion through the newly formed defects and porosities, resulting in a further deterioration of the mechanical properties [10].

A limited extent of water sorption by the composite is considered desirable because it results in a material expansion that can partially compensate for polymerization shrinkage, reducing internal stresses and interfacial strains [11]. However, excessive water sorption accelerates hydrolytic processes that degrade the mechanical, chemical, and biological properties of the composite [10]. Under conditions of high water sorption, functional fillers are dissolved more rapidly, while the polymer network is cleaved into degradation products with lower molecular mass, which are released from the restoration and may compromise the biocompatibility [12]. Swelling and degradation of the polymer matrix in conjunction with the dissolution of reactive fillers may affect the hardness of the composite surface [13]. While being generally considered a determinant of material abrasion resistance, hardness indirectly affects bacterial adhesion, as surfaces that are more susceptible to abrasion are more easily roughened. In this sense, maintaining hardness throughout the life of the restoration is important for the marginal continuity and vertical dimension of the restoration, as well as for limiting bacterial accumulation and the associated risk of secondary caries. In addition to the undesirable degradation processes, a positive consequence of water exposure is the re-precipitation of dissolved calcium and phosphate ions in the form of apatite on the material surface [14]. The ability to precipitate apatite is related to the reactivity of BG, as more reactive BG formulations dissolve faster, making more calcium and phosphate ions available in solution and forming a denser silica-gel layer on the material surface which facilitates re-precipitation [15].

Water-induced degradation is particularly pronounced in composites functionalized with conventional BG 45S5 due to its reactivity and high dissolution rate [16]. To overcome the drawbacks associated with the high reactivity of BG 45S5, a less reactive, low-Na F-containing BG was formulated and investigated as a functional filler for resin composites. In previous studies with experimental composites functionalized with this low-Na F-containing BG, positive results were obtained for the following properties: acid neutralization [17], degree of conversion and polymerization kinetics [18], polymerization shrinkage properties [19], flexural properties [20], ion release [21], and the resulting anti-demineralizing protective effect on enamel [22] and dentin [23]. The aim of the present study was to complement these investigations by evaluating properties related to the exposure of composite materials to water, namely microhardness, mass changes during long-term immersion, water sorption and solubility evaluated according to ISO 4049, and surface precipitation of calcium phosphate.

The research hypothesis of the present study was that functionalizing experimental resin composites with 5–40 wt% of the customized low-Na F-containing BG instead of the conventional BG 45S5 would improve microhardness and reduce water sorption/solubility while maintaining the ability to precipitate calcium phosphate. Therefore, four null hypotheses were formulated assuming that there would be no effect of:

(I). BG amount and type on microhardness;
(II). artificial aging on microhardness;
(III). BG amount and type on long-term water sorption and solubility; and
(IV). BG amount and type on calcium phosphate precipitation.

## 2. Materials and Methods

### 2.1. Preparation of Experimental Resin Composites

BG 45S5 and a low-Na F-containing BG were prepared by the melt-quench method and ground to a particle size of d50 = 3 μm [17]. Both BG types had theoretical network connectivity of 2.1. Network connectivity is a parameter for predicting the degradation of BGs and is determined by the number of network modifiers that disrupt the silicate network by forming non-bridging oxygen bonds. A more disrupted network is less stable and more degraded upon contact with water. For BGs prepared by the melt-quench route, the network connectivity can be calculated based on their composition [15]. In the present study, the composition of the low-Na F-containing BG was adjusted to maintain the same network connectivity as the conventional BG 45S5.

Silanized barium glass and silica were used as reinforcing (inert) fillers. The composition of the reactive and inert fillers used for the preparation of the experimental composites is listed in Table 1.

The experimental resin composites were prepared with 5–40 wt% of one of the two BG types (Table 2), designated as C-series (with BG 45S5) and F-series (with the low-Na F-containing BG). Reinforcing fillers, i.e., silanized barium glass and silica (2:1 wt/wt), were added up to a total filler amount of 70 wt%. The “Control” composite contained only 70 wt% inert silanized barium glass and silica.

The resin system for the experimental composites was prepared by mixing bisphenol A glycidyl dimethacrylate (bis-GMA, Merck, Darmstadt, Germany) and triethylene glycol dimethacrylate (TEGDMA, Merck) in a 60:40 weight ratio. The properties of methacrylate resins are shown in Table 3. The resin mixture was photosensitized by adding 0.2 wt% camphorquinone (Merck) and 0.8 wt% ethyl 4-(dimethylamino)benzoate (Merck). The obtained comonomer mixture and photoinitiator system were homogenized with a magnetic stirrer for 48 h. The photosensitized resin system was mixed with the inorganic fillers in a centrifugal mixer (Speed Mixer TM DAC 150 FVZ, Hauschild & Co., Ltd., KG, Hamm, Germany) at 2000 rpm for 5 min. After mixing, the composites were stored under a vacuum for 48 h to remove air bubbles.

### 2.2. Microhardness Testing

The test specimens of experimental composites (2 × 2 × 16 mm) were fabricated in Teflon split-molds, covered with polyethylene terephthalate film (PET), pressed with two 5-mm-thick glass plates to remove excess material, and light-cured with the Bluephase PowerCure LED curing unit (Ivoclar Vivadent, Schaan, Liechtenstein). The curing unit delivered a radiant exitance of 1050 mW/cm^2^, which was measured with a NIST-referenced UV-vis spectrophotometer system (MARC; BlueLight Analytics, Halifax, NS, Canada). The experimental composite specimens were illuminated with three exposures (20 s each) that overlapped no more than 1 mm with the previously irradiated section. To achieve a maximum attainable cure, the specimens were additionally illuminated from the opposite side in the same manner. Careful grinding with a P4000 silicon carbide paper under running water was used to remove the resin-rich layer from the specimen surface. Specimen surfaces were then polished to a high gloss using a 0.05-μm aluminum oxide suspension and a polishing cloth. For each experimental material, a total of 24 specimens were prepared and divided into three groups (*n* = 8 per group), which were subjected to the following artificial aging protocols:

(I). aging in distilled water at 37 °C for 1 day;
(II). (aging in distilled water at 37 °C for 30 days;
(III). aging in distilled water at 37 °C for 30 days, followed by thermocycling (10,000 cycles between 5–55 °C, dwell time: 30 s) which simulates 1-year aging [24].

After completion of artificial aging, Vickers microhardness of specimen surfaces was measured using a digital microhardness tester (CSV-10; ESI Prüftechnik GmbH, Wendlingen, Germany) with a load of 100 g and a dwell time of 15 s. Vickers microhardness was calculated automatically by the instrument software according to the expression VH = 0.1891 × F/d^2^, where VH = Vickers hardness, F = load (N), d = indentation diagonal length (mm). Five replicate measurements were made on each specimen at the center of the rectangular surface in a 5 mm line along the long axis of the specimen. A mean value was calculated from these five repetitions and used as the statistical unit.

### 2.3. Water Sorption and Solubility Testing

Water sorption and solubility were measured using a gravimetric method according to the modified ISO 4049 protocol [25]. Composite specimens were prepared in cylindrical Teflon molds (diameter = 6 mm, height = 2 mm). The mold openings were covered with PET films and pressed between two glass slides to remove excess material. Specimens were light-cured using the Bluephase PowerCure curing unit with a radiant exitance of 1050 mW/cm^2^ for 20 s on each side of the specimen. Specimen diameter was measured with a digital caliper in two perpendicular planes, while specimen thickness was measured at the center and four equally spaced points along the circumference. These data were used to calculate specimen volume to express water sorption and solubility in µg/mm^3^. Composite specimens (*n* = 6 per experimental group) were placed in a desiccator with silica gel and weighed every 24 h using an analytical balance (MS NewClassic, Mettler Toledo AG, Switzerland) until the difference between two consecutive measurements fell below 0.1 mg, which was considered as reaching a constant mass. This value represented the initial mass (m1). Specimens were individually immersed in closed vials filled with 5 mL of distilled water and stored at 37 °C. The conical bottom of the vials ensured that only the rim of the specimen was in contact with the vial, hence maximizing exposure of the specimen surface to water. After 1, 3, 7, 14, 21, 28, 35, 42, 63, 180, and 360 days, the specimens were removed from the water and carefully blotted with cellulose wipes until they were free of visible moisture, as described in the protocol ISO 4049, and weighed to measure m2(t), which represents sample mass as a function of time after the onset of immersion. Distilled water was replaced every seven days throughout the 360-day observation period. After 360 days, specimens were transferred to the desiccator with silica gel and weighed every 24 h until a constant mass (defined as a mass variation of less than 0.1 mg) was reached, which was noted as the final mass after drying (m3).

The mass change as a function of immersion time was calculated as Δm(t) = m2(t) − m1. Water sorption and solubility in µg/mm^3^ were calculated according to the ISO 4049 equations:

water sorption = (m2(360 days) − m3)/V;

solubility = (m1 − m3)/V;

where m2(360 days) denotes mass at the end of the observation period, and V denotes specimen volume.

### 2.4. Scanning Electron Microscopy (SEM) and Energy Dispersive X-ray Spectroscopy (EDS)

For SEM and EDS examination of the specimen surface after immersion in phosphate-buffered saline (PBS), cylindrical specimens (diameter = 6 mm, height = 2 mm) were prepared as described for water sorption and solubility measurements. Three specimens per material were prepared (*n* = 3), and each specimen was separately immersed in 10 mL of PBS at 37 °C for 3 months. After immersion, the specimens were rinsed thoroughly with deionized water, dried in a desiccator, and examined as prepared (unpolished and unsputtered) using a scanning electron microscope equipped with an EDS detector (SEM, Quanta FEG 400, FEI Company, Eindhoven, The Netherlands) in low vacuum at a voltage of 5 kV. EDS spectra were collected from three randomly chosen areas on each specimen in order to obtain a representative average spectrum.

### 2.5. Fourier-Transform Infrared Spectroscopy

Fourier- transform infrared spectroscopy (FTIR) analysis was performed using the same specimens (*n* = 3) that were previously immersed for 3 months in PBS and used for SEM/EDS analysis. FTIR spectra of the specimen surface were acquired using the attenuated total reflectance (ATR) diamond accessory of the Nicolet iS50 FTIR spectrometer (Thermo Fisher Scientific, Waltham, MA, USA). Thirty scans per spectrum were acquired in absorbance mode in a spectral range of 3500–400 cm^−1^ and a resolution of 4 cm^−1^. Calcium phosphate was identified from the spectral bands at 560 and 600 cm^−1^ assigned to PO_4_ bending [14].

### 2.6. Statistical Analysis

Sample size estimation based on the preliminary measurements was performed using G*Power software [26] and revealed that the required sample size to detect a 15% difference with statistical power greater than 0.8 was *n* = 8 for microhardness and *n* = 6 for water sorption/solubility.

After checking for normality of distribution using the Shapiro-Wilk test and normal Q-Q plots, data were analyzed parametrically using analysis of variance (ANOVA). For microhardness, a mixed model two-way ANOVA indicated a significant interaction between the factors “material” and “aging protocol,” so the analysis was followed by one-way ANOVAs to determine isolated effects of each factor at fixed levels of the other factor. Comparisons of microhardness values among the materials were made using one-way ANOVA with Tukey’s post-hoc tests for multiple comparisons, whereas comparisons among aging protocols within the same material were considered repeated observations and were therefore made using repeated measurements ANOVA with Bonferroni adjustment for multiple comparisons. Water sorption and solubility data were compared among materials using one-way ANOVA with Tukey’s post-hoc adjustment. The overall significance level was set at 0.05 for all analyses. Statistical analysis was performed using SPSS (version 25; IBM, Armonk, NY, USA).

## 3. Results

**Figure 1:**
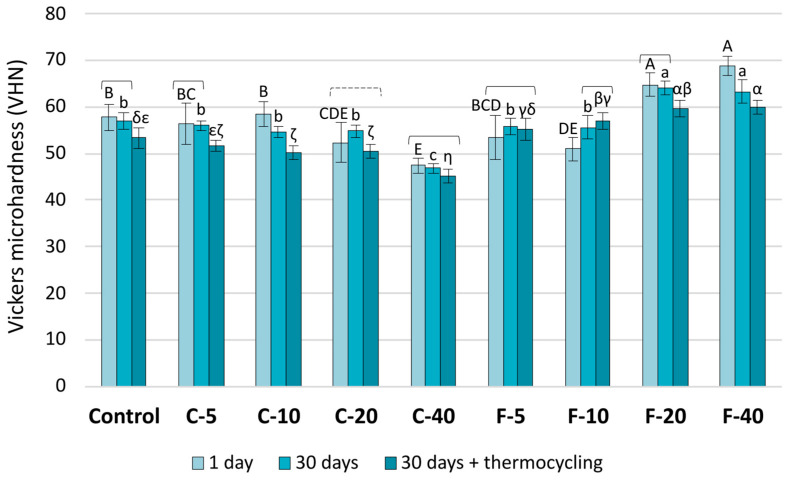
Vickers microhardness presented as mean values ± SD. Square brackets indicate statistically similar values among artificial aging protocols. The dashed square bracket indicates statistically similar values of the first and the last time point, which were both significantly different from the middle time point. Statistically similar values among materials are denoted by the same uppercase, lowercase, and Greek letters for the following artificial aging protocols, respectively: 1 day, 30 days, and 30 days + thermocycling.

Microhardness values decreased significantly with artificial aging for all materials except C-40 and F-5. Within the C-series, a significant microhardness decrease was observed due to increasing amounts of BG (Figure 1). The 1-day microhardness values in the C-series were significantly reduced at 20 wt% or more of BG compared to the Control material, while at the final time point of artificial aging, a statistically significant microhardness reduction in the C-series began already with 10 wt% of BG. For the F-series, 1-day measurements showed that 5 wt% of BG resulted in statistically similar microhardness values to that of the Control, 10 wt% resulted in a significant reduction, while higher BG amounts (20 and 40 wt%) led to a microhardness improvement compared to the Control. The microhardness values at the last time point of artificial aging in the F-series were statistically similar to that of the Control for 5 wt% of BG, while at all other BG amounts (10–40 wt%), microhardness values were significantly higher compared to the Control.

**Figure 2:**
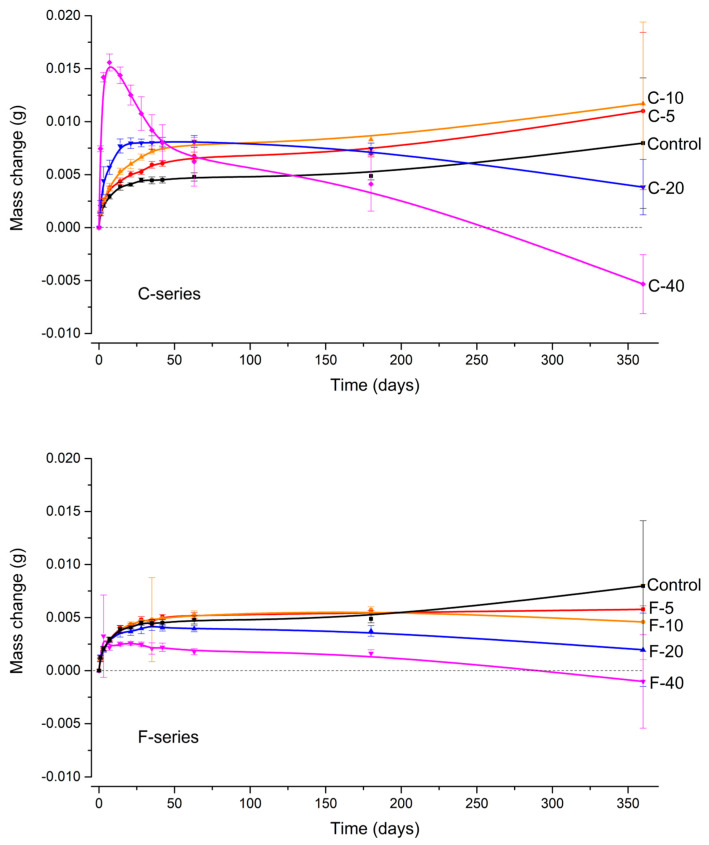
Mass changes (mean values ± SD) during aqueous immersion of experimental composites. The C-series and the F-series are shown in separate panels to avoid curve overlap. The mass change curve for the Control composite is shown in both panels to allow direct comparisons.

The mass change during immersion in water as a function of time is shown in Figure 2. The Control composite, C-5, and C-10, showed a continuous mass increase throughout the one-year observation period. In contrast, the curves for C-20 and C-40 showed inflection points, after which the mass decreased with time. While the mass for C-20 was positive at the end of the observation period, the steep drop in the curve for C-40 resulted in an overall negative mass change.

Compared to the Control composite, C-5 and C-10 had a higher mass increase, while C-20 and C-40 had a lower final mass. In contrast, all F-series composites showed a lower mass increase compared to the Control composite. While F-5 and F-10 exhibited stable mass at the end of the observation period, F-20 and F-40 showed similar behavior to the materials with the corresponding BG amounts from the C-series, i.e., an inflection point followed by a mass decrease. F-40 also exhibited a slight negative mass change, but not as pronounced as the corresponding C-series material.

**Figure 3:**
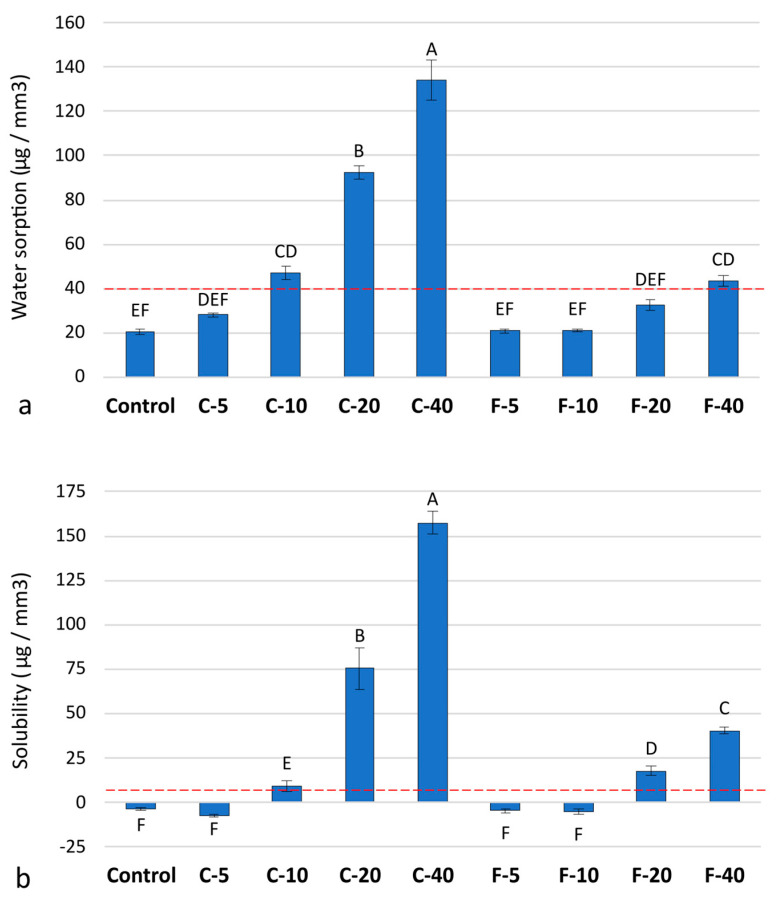
Water sorption (**a**) and solubility (**b**) in μg/mm^3^ presented as mean values ± SD. The same uppercase letters denote statistically similar values among materials. Red dashed lines denote the ISO 4049 thresholds of 40.0 and 7.5 μg/mm^3^ for water sorption and solubility, respectively.

Water sorption increased with higher amounts of BG in both the C-series and F-series (Figure 3a). The increase in water sorption was more pronounced in the C-series, which showed up to a 7-fold increase between C-40 and the Control, whereas the corresponding increase in the F-series (F-40 vs. Control) was only 2-fold. Statistical comparisons among materials in the C-series showed that a significant increase in water sorption over the Control material was measured at as little as 10 wt% BG, while 40 wt% BG was required for the F-series to show a significant increase in water sorption over the Control material.

Solubility increased with higher BG amounts, with an abrupt increase noted for C-20 and C-40 (Figure 3b). Negative solubility was measured for the Control material and three materials with low BG amounts (C-5, F-5, and F-10).

**Figure 4:**
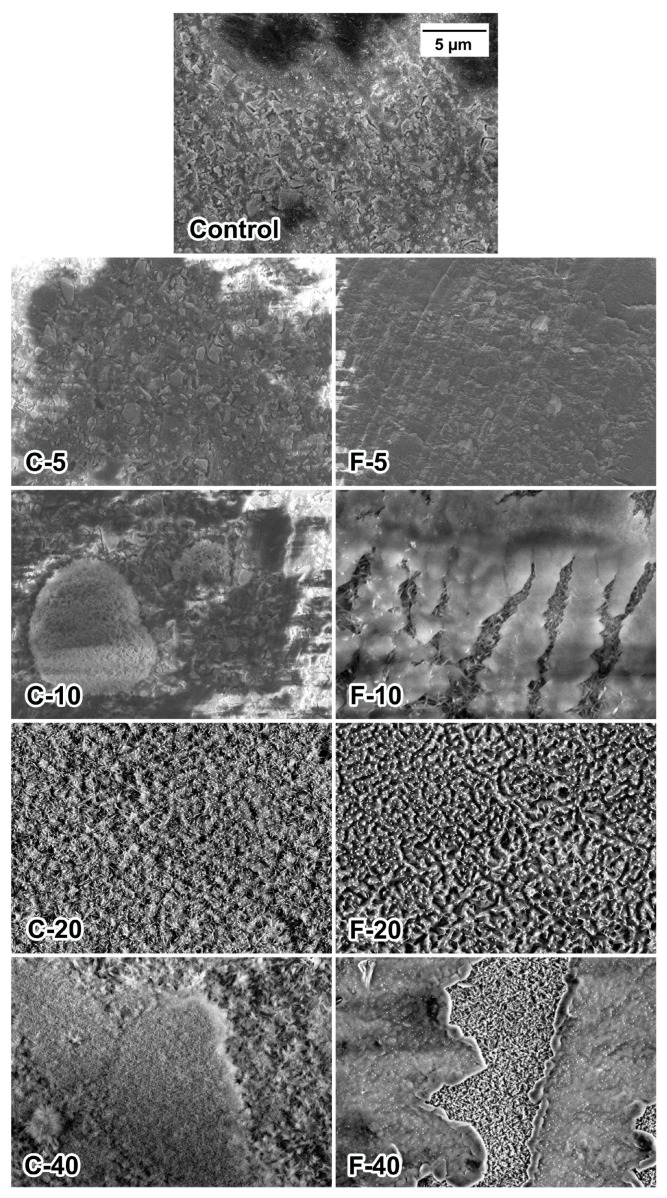
Scanning electron microscopy images of experimental composite surfaces after 3 months of immersion in phosphate-buffered saline.

SEM images of the composite surface after 3 months of PBS immersion are shown in Figure 4. The Control composite and the materials with 5 wt% BG showed a microstructure typical of resin composites, i.e., irregular glass particles embedded in resin. No surface precipitates were identified in these composites. For C-10, the precipitate of dense, plate-like crystals was localized in irregular agglomerates, while for F-10, the precipitate was needle-like and formed below surface cracks in the resin-rich layer. Materials C-20 and F-20 exhibited uniform coverage of their surface with needle-like crystals. For C-40, complete coverage was also observed, but with areas of varying density. For F-40, the dense precipitate formed in the cracks of the resin-rich layer, while the surface of the intact resin-rich layer did not form any precipitate.

**Figure 5:**
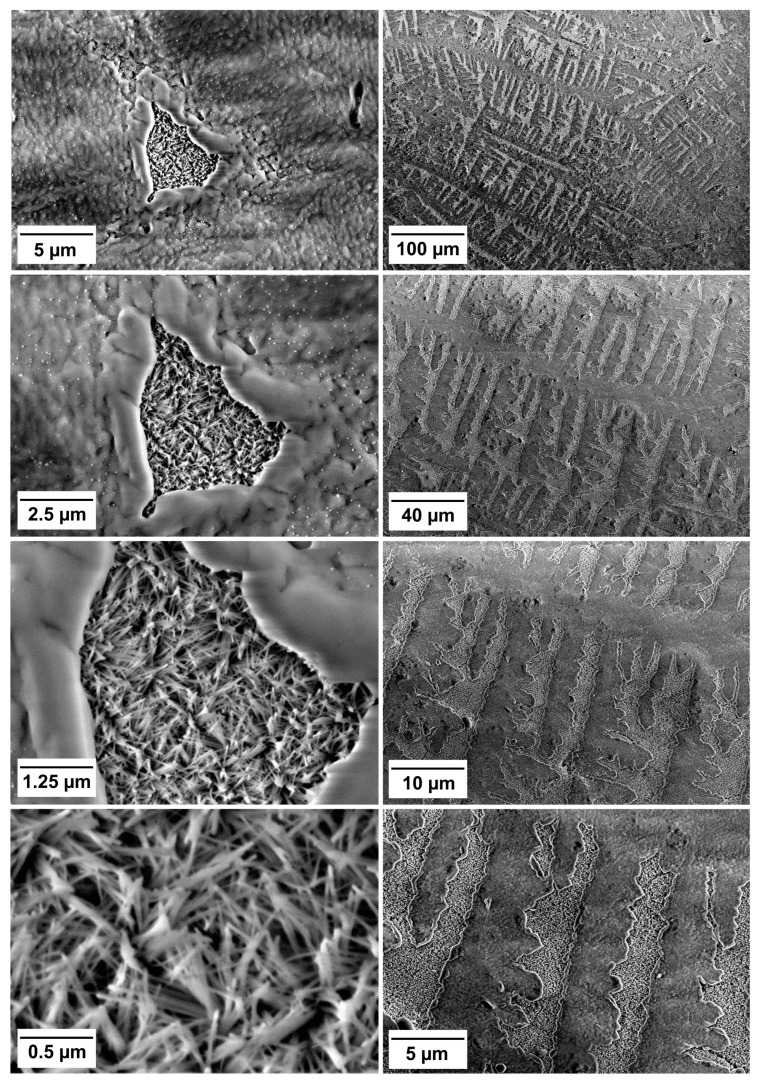
Scanning electron microscopy images of the material F-40 after 3 months of immersion in phosphate-buffered saline. The increasing magnifications of the same area show the formation of precipitate inside a solitary surface defect (**left** column) and in multiple defects following the pattern of surface microdamage due to specimen preparation (**right** column).

The tendency of F-40 to form the precipitate preferentially in the defects of the resin-rich layer is shown in Figure 5. Magnifications increase from top to bottom panels. Lower magnifications show the localization of the precipitate in individual cracks (left column) or the pattern of surface defects formed by tearing off portions of the resin-rich layer with a polyethylene terephthalate strip during specimen preparation (right column). Higher magnifications show the needle-like morphology of the precipitate.

**Figure 6:**
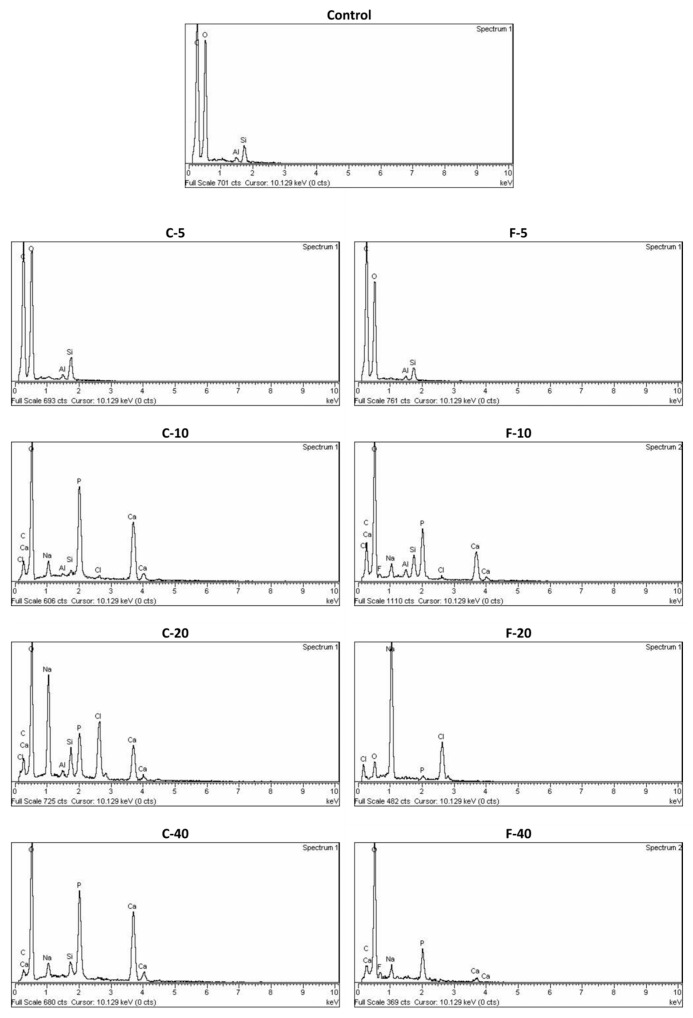
Energy dispersive X-ray spectra of experimental composite surfaces after immersion in phosphate-buffered saline.

EDS spectra are shown in Figure 6. The main elements detected on the surface of the Control material (carbon, oxygen, aluminum, silicon) originated from the resin matrix and glass fillers. The materials with low BG amounts (C-5 and F-5) showed similar EDS spectra to the Control material, while at 10 wt% BG, calcium and phosphorus were detected in the precipitate along with sodium and chlorine. Increasing the BG content to 20% resulted in even higher amounts of sodium and chlorine for C-20, while for F-20, sodium and chlorine dominated the EDS spectra. Compared to the composites with 20 wt% BG, the materials with the highest BG content (40 wt%) had lower relative amounts of sodium and chlorine and detectable amounts of calcium and phosphorus. The calcium and phosphorus signals were more pronounced in C-40 than in F-40. Signs of fluorine in the EDS spectra were found in F-10 and F-40.

The calcium-to-phosphate ratio quantified from the EDS spectra is presented in Table 4. The Ca/P ratios ranged between 1.01 and 1.46.

**Figure 7:**
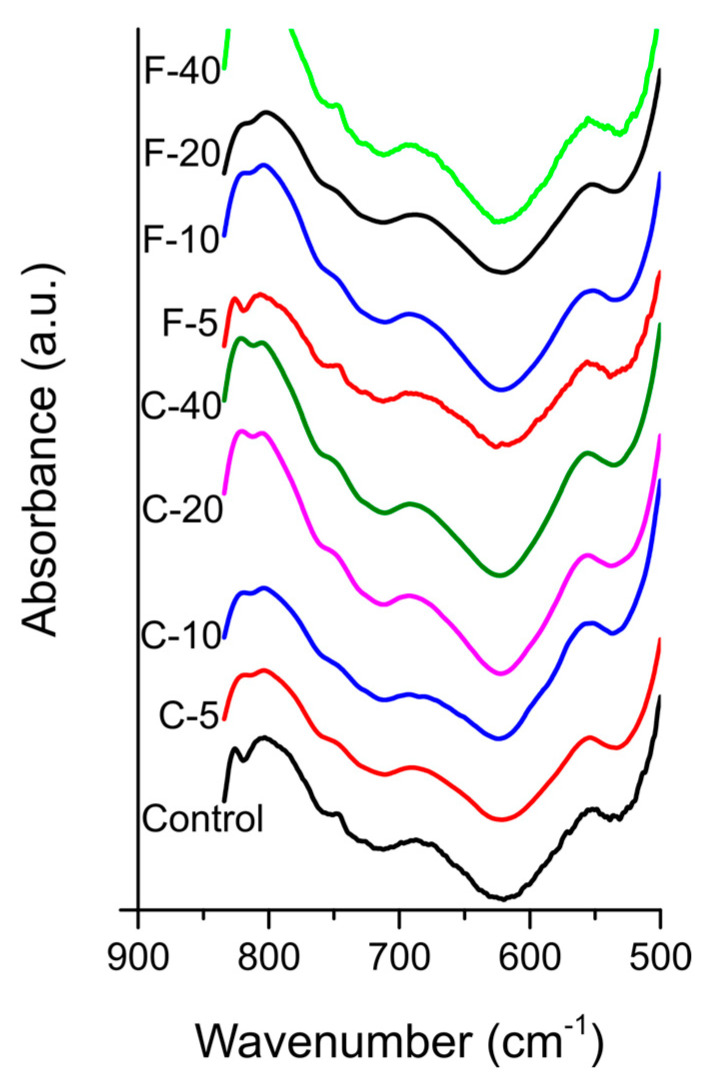
Fourier-transform infrared spectra of experimental composite surfaces before immersion in phosphate-buffered saline.

**Figure 8:**
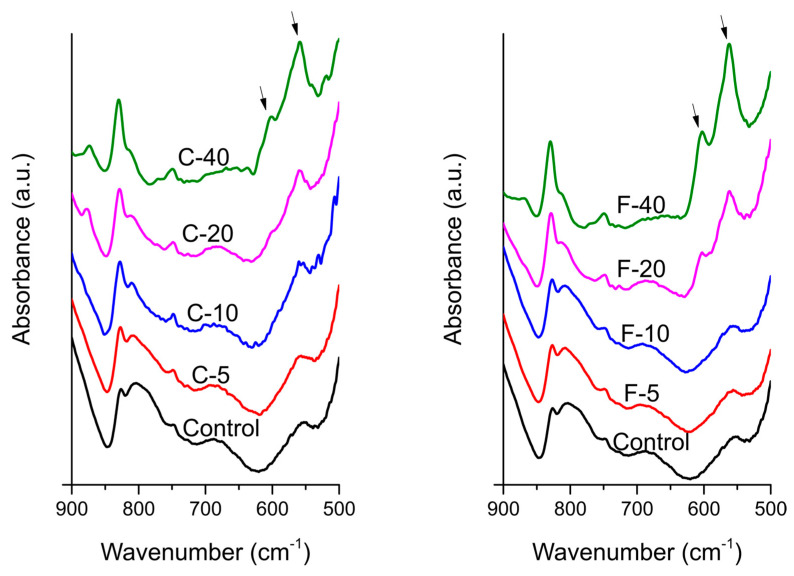
Fourier-transform infrared spectra of experimental composite surfaces after immersion in phosphate-buffered saline. The bands at 560 and 600 cm^−1^ (PO_4_ bending) are marked by arrows.

The FTIR spectra collected before PBS immersion (Figure 7) did not show phosphate spectral bands at 560 and 600 cm^−1^. In FTIR spectra collected after PBS immersion (Figure 8), the spectral bands at 560 and 600 cm^−1^, indicating calcium phosphate precipitation on the specimen surface, were clearly visible for the materials containing 20 and 40 wt% of both BG types, while at lower BG amounts the corresponding part of the spectrum was indistinguishable from that of the Control material.

## 4. Discussion

This study investigated the water-induced changes in the properties of experimental composites functionalized with two BG types. It was found that the customized low-Na F-containing BG resulted in improved microhardness and lower water sorption/solubility compared to the conventional BG 45S5, while the ability to precipitate calcium phosphate was present in the composites functionalized with both BG types. The null hypotheses I-III were rejected because there were statistically significant effects of the amount and type of BG on microhardness, whereas artificial aging significantly affected the degradation of microhardness. The fourth null hypothesis, which assumed that calcium phosphate precipitates would not form after immersion in PBS, was rejected for composites with a BG filler content of 10 wt% and above.

Similar to other mechanical properties of resin composites, microhardness is also gradually degraded during material aging in aqueous environments. The degradation of microhardness is associated with several processes, including polymer swelling and plasticization, dissolution of filler particles, and weakening of the filler/resin interface [10]. Since all these processes depend on water uptake, material degradation can be controlled by limiting the hydrophilicity of the resin composite [27]. This has been applied to commercial composites that can maintain favorable mechanical properties due to their hydrophobic character. On the other hand, remineralizing composites are intentionally made more hydrophilic to allow water diffusion and ion release. Hydrophilicity also increases with the aging of the material, as reactive fillers dissolve and form porosities and water channels in the material structure. Therefore, ion-releasing composites are not only more hydrophilic from the outset, but their water permeability additionally increases over time as they are exposed to water.

Considering the microhardness changes that occurred during artificial aging, a 7.6% reduction in microhardness was observed for the Control composite, while BG-functionalized composites showed a 3.6–14.1% reduction in microhardness, except for F-10, which showed a statistically significant microhardness improvement of 11.7%. When the effect of different amounts of BG between 5–40 wt% is expressed as a relative microhardness reduction compared to the Control composite, the values were 1.6–17.9%. An exception was observed for the materials with a high amount of the low-Na F-containing BG (F-20 and F-40), which showed a statistically significant improvement (11.9–19.0%) of microhardness compared to the Control material. The relative magnitude of this improvement may even offset the aforementioned aging-related deterioration of microhardness (3.6–14.1%). Such an extensive microhardness improvement for F-20 and F-40 compared to the Control composite cannot be attributed solely to the higher stability of the customized BG, as it is unlikely that a BG composition would improve the micromechanical properties better than inert silanized fillers. Instead, the improvement is likely caused indirectly due to the ability of the customized BG to improve the degree of conversion of the polymer matrix compared to the Control material. This aspect was investigated in a previous study, which showed that the degree of conversion was reduced from 67.4% measured for the Control composite to 63.7% when 40 wt% BG 45S5 was added but improved to 71.6% when 40 wt% of the customized BG was added [20]. In that study, [20], an improved degree of conversion resulted in improved macro-mechanical properties (flexural strength and modulus), while in the present study, an analogous effect was observed on micromechanical properties (microhardness).

In contrast to the aging-induced degradation of microhardness, which was observed for other experimental materials in our study, microhardness for F-10 increased significantly with aging. The possibility that microhardness changes in either direction with simulated aging was reported in a study by Sauro et al. [28] for experimental adhesive systems functionalized with BG 45S5 or with zinc-modified BG. In that study, the adhesive filled with BG 45S5 showed a microhardness reduction (from 22.3 to 13.9 KHN) during the 60-day artificial aging, while the adhesive filled with a zinc-modified BG initially reached a lower microhardness (18.3 KHN), which increased to 29.8 KHN with aging [28]. An increase in microhardness with short-term aging (21 days) was also reported for adhesive resins functionalized with 10–20 wt% BG 45S5 [29]. In general, the direction of the aging-dependent change in microhardness depends on the relative contributions of material degradation due to water sorption and the continuation of the post-cure polymerization [30]. When the effect of post-cure polymerization predominates over degradation, the net result is an increase in microhardness. The overall extent and kinetics of post-cure polymerization depend on many factors, including the reactivity of the resin, the viscosity of the composite, the size and loading of the filler particles, and the initial extent of polymerization [31]. In the case of BG-containing composites in our study, both the short-term degree of conversion achieved during exposure to the curing light and the slower long-term post-cure increase in the degree of conversion can be additionally influenced by the interaction of BG with free radical-mediated polymerization. Regardless of other factors, the addition of BG can retard the polymerization reaction with or without reducing the final degree of conversion [32]. Therefore, the continuation of post-cure polymerization, further favored by the enhanced mobility of the reactive species in the “hot” part of the thermal cycle (55 °C), is a likely explanation for the statistically significant microhardness increase observed for F-10.

Other studies on BG-functionalized resin composites, using different resin systems, BG types, and BG amounts, reported mixed effects of the inclusion of BG fillers on the microhardness of the composite. Although in most cases, microhardness was reduced by the addition of BG fillers [5,33,34,35,36], in some cases, microhardness was improved [37,38]. In experimental composites based on a proprietary resin matrix, the replacement of 23% of the reinforcing barium glass fillers with BG 45S5 resulted in a reduction of microhardness of the composite by about 50% compared to the composite containing only reinforcing fillers [33]. A similar result was observed when up to 5.4 wt% zinc-doped phosphate-based glass was added to a commercially available flowable composite, reducing microhardness by about 12% compared to the control material with reinforcing fillers only [34]. For experimental Bis-GMA/UDMA-based resin composites developed as orthodontic adhesives, different effects on microhardness were reported, depending on the BG type. For example, the addition of up to 20 wt% BG 45S5 resulted in a significant decrease in microhardness (approximately 23% less compared to the control), whereas the addition of the same amount of niobium phosphate-based bioactive glass had no significant effect on microhardness [35]. The BG type was also a factor in a study of filled experimental adhesives, which showed higher susceptibility to ethanol softening when modified with 2 wt% BG 45S5 compared with the same amount of niobium-modified BG and unfilled control adhesive [39]. The type of resin to which BG fillers were added was also shown to affect the microhardness; in a study of a commercial adhesive system functionalized with 10–20 wt% BG 45S5, a slight (statistically non-significant) reduction in microhardness was reported with the addition of BG [29], while the addition of the same amount, type and particle size of BG to an epoxy resin significantly increased microhardness [37], indicating a possible interaction between BG and the epoxy matrix that does not appear to occur with a methacrylate matrix. A study by Hyun et al. [36] reported that the addition of two types of customized BG resulted in a reduction of microhardness compared to the control material filled with reinforcing fillers only and also highlighted that pure chemical degradation by the aqueous environment was more influential for the deterioration of microhardness with aging than the presence of a Streptococcus mutans biofilm. A study by Odermatt et al. [5] showed that the addition of 15 wt% BG 45S5 to a commercial flowable composite significantly reduced microhardness regardless of the particle size of BG (nano- or micro-sized fillers). In contrast, replacing 10 wt% of nano-sized silica fillers with the same amount of copper-doped mesoporous BG nanoparticles improved microhardness by 35.9% [38], possibly indicating that the porosity of the nanoparticles improves filler/resin interlocking. However, after 28 days of artificial aging in distilled water, the improvement was only 9.0%, indicating that the positive effects of the interaction between the mesoporous BG particles and the resin were likely temporary. Overall, several factors were shown to play a role in determining the microhardness of BG-functionalized composites and their changes over time. The experimental composites in the present study were designed to resemble a conventional nano-hybrid composite based on a hydrophobic Bis-GMA/TEGDMA resin matrix in terms of resin type, filler amount, and filler particle size. All other factors being equal, the customized BG showed a significant improvement in microhardness compared to BG 45S5. Moreover, the difference in microhardness between the composites functionalized with BG 45S5 and the customized BG became more pronounced as the BG amount in the composites increased.

The lack of a hydrophobic coating on the surface of the reactive BG fillers is a major reason for the high water sorption of the experimental composites. This problem can theoretically be mitigated by coating the BG particles with a silane layer to make them less hydrophilic, as shown by Oral et al. [40], who significantly reduced the water sorption of the experimental composites by silanizing BG S53P4 in a resin system of methyl methacrylate and ethylene glycol dimethacrylate (95/5 wt%). Similarly, water sorption was successfully reduced by surface silanization of BG 45S5 and two types of alumina-doped BG [41]. If the reduction of water sorption were the only goal in the development of BG-functionalized composites, surface silanization would be an effective solution. However, the silanization of reactive particles may interfere with their ion release, which is why these fillers are usually used without silanization [3,16,29]. In addition, some experimental ion-releasing composites are intentionally designed to be extremely hydrophilic by containing large amounts of the monomer HEMA to enhance water diffusion and ion release. In a series of such experimental composites based on a highly hydrophilic Bis-GMA/HEMA resin system (60/40), even the negative control material (which consisted of only 70 wt% silanized inert fillers) exhibited a high water sorption of 67.6 μg/cm^3^, exceeding the maximum value of 40 μg/cm^3^ established by ISO for restorative resin composites [41]. When the same resin system was functionalized with 10 wt% of three different BG compositions, even higher water sorption was measured (107.4–132.7 μg/cm^3^). Such a highly hydrophilic resin system was not required for experimental composites in our study because the hydrophilicity of the unsilanized BG particles themselves allowed sufficient water diffusion, as demonstrated in previous in vitro studies of the same experimental composites regarding ion release [21,42] and protective effects on dental hard tissues [22,23].

It is common for ion-releasing composites to exceed ISO 4049 limits for water sorption and solubility, as these requirements have historically been defined exclusively for non-releasing restorative composites. For this group of composites, high values for water sorption and solubility usually indicate negative material behavior, implying dimensional/chemical/mechanical instability and the release of toxic substances. In contrast, for remineralizing composites, a certain level of water sorption and solubility is a necessary prerequisite and also a consequence of the ion-releasing activity. Even small amounts of reactive fillers are sufficient to exceed the threshold values of ISO 4049, as shown for experimental orthodontic adhesives with 10 wt% Sr-doped BG nanoparticles in combination with monocalcium phosphate monohydrate in a 1:1 ratio [43] and for fissure, sealants functionalized with 12.5 wt% BG 45S5 in a Bis-GMA/TEGDMA (1:1) resin system [44]. In our study, the ISO 4049 threshold for water sorption was exceeded in the C-series for composites with BG amounts of 10 wt% or more, while all composites in the F-series showed water sorption below the ISO 4049 threshold. Only for the highest amount of the customized BG in F-40 was water absorption observed near the ISO 4049 threshold (43.5 μg/cm^3^); however, a comparison of this value with the threshold of 40 μg/cm^3^ showed no statistical significance. A comparison of water sorption values between the C-series and the F-series shows that the composites with BG 45S5 have higher water sorption values by 33.5%, 122.5%, 183.9%, and 208.3% compared to the customized BG for reactive filler amounts (wt%) of 5, 10, 20, and 40, respectively. This is in agreement with other studies, which also showed that methacrylate resins functionalized with modified BG formulations exhibited lower water sorption than the 45S5 formulation [45,46] since the latter was originally developed for use in orthopedics and is known for its high reactivity and solubility. Namely, a study by Raszewski et al. [45] compared the water sorption and solubility of methyl methacrylate resin with 10 wt% of different BG (45S5, S53P4, an F-modified BG, and a Cl-modified BG) and found that the statistically highest sorption and solubility occurred for composites containing BG 45S5. Another study by Sfalcin et al. [46] on experimental enamel resin infiltrants Bis-EMA/TEGDMA (25:75) functionalized with 10 wt% of various reactive fillers found that BG 45S5 and amorphous calcium phosphate exhibited the significantly highest water sorption compared to other reactive fillers, including hydroxyapatite, zinc-modified BG, calcium silicate and tricalcium phosphate.

In conventional composites, solubility has traditionally been equated with the undesirable and potentially harmful release of residual monomers [10]. In addition, inert glasses have recently been shown to degrade in water to a greater extent than previously thought, potentially releasing considerable amounts of barium, silicon, boron, aluminum, and other constituents [47]. Unlike conventional composites, solubility values for ion-releasing composites calculated according to ISO 4049 reflect the cumulative release of both undesirable substances and therapeutic ions (e.g., calcium, phosphate, and fluoride). Dissolution of BG also releases other ions, such as sodium, which have no harmful or therapeutic effects due to their low concentrations in the oral cavity. Although the ISO 4049 gravimetric method does not distinguish between the types of species released, the significantly lower solubility of the F-series compared to the C-series suggests better dimensional stability of the experimental composites functionalized with the customized BG. Although the F-series has lower solubility, it was shown in a previous study [21] to release therapeutic ion concentrations sufficient to protect dental hard tissues from demineralization in vitro [22,23]. The higher solubility of the C-series compared to the F-series may be primarily attributed to the higher reactivity of BG 45S5, while the negative effect on resin polymerization caused by high amounts of BG 45S5 [32] may also have contributed to the increase in solubility, which is particularly evident from an abrupt increase in solubility for materials C-20 and C-40. On the other hand, some materials (Control, C-5, F-5, and F-10) showed negative solubility, a phenomenon suggesting that some of the absorbed water is no longer extractable because it is either bound to the resin or reacted with filler particles to form hydroxides [48,49]. Since the negative solubility values for C-5, F-5, and F-10 were statistically similar to those of the Control material, the retention of non-extractable water cannot be directly attributed to the BG fillers but seems to occur within the “conventional” components of the materials, i.e., the resin system and the reinforcing fillers. The positive solubility in other materials reflects the fact that the modest effect of water retention was exceeded by a much larger mass loss of the materials.

SEM and EDS results indicate that the composites with BG amounts of 10–40 wt% formed calcium phosphate precipitates after immersion in PBS. Compared to SEM/EDS, FTIR analysis was less sensitive and suggested the formation of calcium phosphate through the identification of phosphate vibrational bands [14,50] only for composites with 20 and 40 wt% BG. On SEM images, the calcium phosphate crystals resembled a needle-like morphology, as previously reported by Zhang et al. for the hydroxyapatite [51]. It is possible that a mixture of fluorapatite and hydroxyapatite was formed by the F-series; however, these two types of calcium phosphate could not be distinguished based on the available data, as a magic angle spinning-nuclear magnetic resonance analysis is required for definitive differentiation [52]. According to the EDS analysis, FTIR patterns, and crystal morphology [14,51,52], the precipitate may be considered calcium-deficient apatite with the following Ca/P ratios: 1.23 (C-10), 1.42 (C-20), 1.46 (C-40), 0.78 (F-10), and 1.01 (F-40). For F-20, the Ca/P ratio was not determined because no Ca signal was detectable. Instead, the EDS spectrum for this material was dominated by Na and Cl peaks, suggesting that the crystalline precipitate is primarily sodium chloride rather than calcium phosphate. While the possibility of an experimental artificial artifact cannot be ruled out, it is also possible that a NaCl layer formed on the calcium phosphate precipitate and therefore dominates the EDS spectrum. This aspect should be investigated further to confirm whether NaCl precipitation was favored over calcium phosphate in the case of F-20. It should be noted that trace amounts of NaCl originating from the PBS solution were detected on the surfaces of other composites with BG amounts between 10–40 wt% but in much smaller amounts than for F-20.

The SEM images indicate that the experimental composites differed in the formation of a resin-rich layer on the specimen surface. This layer, with a lower filler content than the bulk of the material, forms due to the attraction of the methacrylate monomers by the PET film covering the specimen surface [53]. For the composites that formed a thin resin-rich layer and no precipitate (Control, C-5, and F-5), the filler particles are clearly visible in the SEM images. On the other hand, two composites (F-10 and F-40) tended to form a markedly thick resin-rich layer that covered the filler particles and prevented the precipitation of calcium phosphate. However, in these materials, the precipitate formed locally in the cracks of the resin-rich layer, as the cracks exposed the filler particles to water and allowed their dissolution and re-precipitation of calcium phosphate. In the clinical application of resin composites, finishing and polishing procedures are commonly employed to remove the resin-rich layer to improve color stability and achieve better mechanical properties of the restoration surface [54]. For the experimental composites that tended to form a thick resin-rich layer (F-10 and F-40), these procedures would also improve calcium phosphate precipitation by exposing BG particles. In any case, the different thicknesses of the resin-rich layer observed in the experimental composites deserve further investigation, as this layer may influence the caries-preventive effect by isolating reactive fillers from the aqueous environment and reducing their dissolution, as well as the release of remineralizing ions [42].

## 5. Conclusions

Based on the results of this in vitro study on the potential of two types of bioactive glass for use as functional fillers in resin composites, a customized low-sodium fluoride-containing bioactive glass showed a comparative advantage over the conventional 45S5 formulation in terms of better microhardness, lower water sorption, and lower solubility. These improvements suggest better mechanical, chemical, and dimensional stability of the composites functionalized with the customized bioactive glass without compromising the potential of the materials to precipitate calcium phosphate.

## Figures and Tables

**Table 1:** Inorganic fillers used in experimental resin composites.

	Bioactive Glass 45S5	Low-Sodium Fluoride-Containing Bioactive Glass	Inert Barium Glass	Silica
Particle size (d50)	3 µm	3 µm	1 µm	5–50 nm
Composition (wt%)	45.0% SiO_2_ 24.5% CaO 24.5% Na_2_O 6.0% P_2_O_5_	33.5% SiO_2_ 33.0% CaO 10.5% Na_2_O 11.0% P_2_O_5_ 12.0% CaF_2_	55.0% SiO_2_ 25.0% BaO 10.0% Al_2_O_3_ 10.0% B_2_O_3_	>99.8% SiO_2_
Silanization (wt%)	none	none	3.2	4–6
Manufacturer	Schott, Mainz, Germany	Schott, Mainz, Germany	Schott, Mainz, Germany	Evonik, Hanau, Germany
Product name/LOT	G018-144/M111473	experimental batch	GM27884/Sil13696	Aerosil R 7200/157020635

**Table 2:** Composition of experimental resin composites.

Material Designation		Filler Composition (wt%)			Total Filler Ratio (wt%)
		Bioactive Glass 45S5	Customized Low-Sodium Fluoride-Containing Bioactive Glass	Reinforcing Fillers (Inert Barium Glass: Silica = 2:1)	
	Control	0	0	70	70
C-series	C-5	5	0	65	70
	C-10	10	0	60	70
	C-20	20	0	50	70
	C-40	40	0	30	70
F-series	F-5	0	5	65	70
	F-10	0	10	60	70
	F-20	0	20	50	70
	F-40	0	40	30	70

**Table 3:** Monomers used in experimental resin composites.

	Bisphenol A-Glycidyl Methacrylate (bis-GMA)	Triethylene Glycol Dimethacrylate (TEGDMA)
CAS Number	1565-94-2	109-16-0
Molecular formula	C_29_H_36_O_8_	C_14_H_22_O_6_
Molar mass (g/mol)	512.60	286.32
Refractive index (at 25 °C)	1.540	1.4595
Viscosity (Pa·s)	910	0.01

**Table 4:** Calcium to phosphorus ratio according to EDS spectra.

Composite Material	Ca/P Ratio
Control	N/A
C-5	N/A
C-10	1.23
C-20	1.42
C-40	1.46
F-5	N/A
F-10	0.78
F-20	N/A
F-40	1.01

## Data Availability

Datasets are available from the corresponding author upon reasonable request.
